# Exposure to non-nestmate odors changes the odorant receptor profile in *Acromyrmex echinatior* leaf-cutting ants

**DOI:** 10.1016/j.isci.2026.116665

**Published:** 2026-07-13

**Authors:** Mélanie Bey, Naomi Jeanne Luna Alex, Lisa Maczkowicz, Yoann Pellen, Joel Vizueta, Volker Nehring

**Affiliations:** 1 Department of Evolutionary Biology and Ecology, Institute of Biology I, University of Freiburg, Freiburg, Germany; 2 Institute of Evolution, Department of Evolutionary and Environmental Biology, University of Haifa, Haifa, Israel; 3 Section for Ecology and Evolution, Department of Biology, University of Copenhagen, Copenhagen, Denmark

**Keywords:** chemoreception, habituation, learning, nestmate recognition

## Abstract

Social insects recognize their nestmates by colony-specific olfactory labels stored as neural templates in the memory. The template is often considered a product of learning in higher brain centers. However, some evidence suggests that the template may be stored in the neural periphery, i.e., the antennae or antennal lobes. We investigated a potential mechanism for peripheral nestmate recognition templates: the composition and plasticity of the antennal odorant receptor (OR) profile. We found that OR gene expression in leaf-cutting ants is colony-specific, mirroring the colony-specific recognition labels. After exposure to non-nestmate odors, the ants exhibited reduced aggression toward the non-nestmate label, indicating habituation. When we exposed two different colonies to the same non-nestmate odor, their OR profiles converged. These results suggest that the olfactory system can adapt to the current nest-specific olfactory environment and may explain how habituation plays a role in nestmate recognition.

## Highlights

• We find that odorant receptor gene expression is colony-specific
• Ants are known to habituate to non-nestmate odors
• Exposing ants to non-nestmate odors predictably changes OR gene expression
• OR profile dynamics may underlie habituation and optimize nestmate recognition

## Graphical abstract

**Figure:**
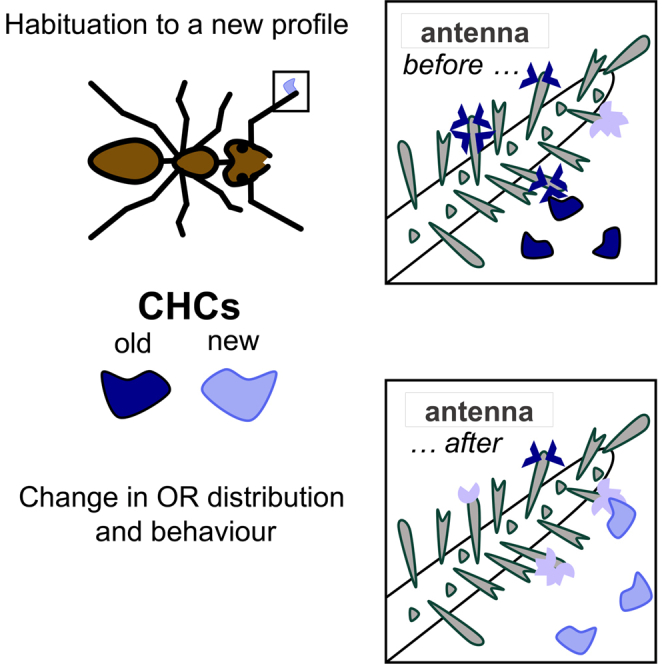

## Teaser

zoology; entomology; neuroscience

## Introduction

Every species perceives the world in its own way. Evolution produced notable examples of specialized perception such as the trichromatic vision in primates,^1^ the remarkable somatosensory organs of the star-nosed mole,^2^ and the fascinating olfaction abilities of the insects.^3^ Insects require perception of a large repertoire of pheromones and other odorants in order to perform species- and mate-recognition, or simply to forage successfully. Insects perceive reality beyond the capacity of the human mind to comprehend^4^ and employ a multitude of sensory receptors, including odorant receptors (ORs). Social insects rely on olfaction to distinguish between nestmates and non-nestmates, a crucial capacity that allows division of labor and provides protection against intruders and parasites.^5^^,^^6^^,^^7^^,^^8^

Nestmate recognition is based on the identification of colony-specific odors (“labels”) that insects bear on their cuticles. The labels are composed of a cuticular hydrocarbon (CHC) mixture, which workers compare to a neural template of their colony’s olfactory identity.^9^ The template can change over time when the colony label changes.^5^^,^^10^^,^^11^^,^^12^ Numerous studies investigated the behavioral aspects of nestmate template dynamics and showed that social insects exposed to non-nestmate odors integrate the non-nestmate labels into their templates, subsequently treating the former strangers as if they were nestmates.^13^^,^^14^^,^^15^^,^^16^^,^^17^^,^^18^ While the behavioral effects are well described, the underlying physiological mechanisms remain elusive. It is unclear whether the template construction and its flexibility is based on sensory adaptation, habituation, or another form of learning. We know that social interactions with nestmates such as trophallaxis or allogrooming,^19^ as well as some forms of associative learning,^20^^,^^21^ are required for template construction and modification. The nature of the template and its location in the nervous system is subject to ongoing debate, giving rise to two hypotheses: memory formation in the central nervous system^8^^,^^22^ versus peripheral adjustments in recognition.^16^^,^^18^^,^^23^^,^^24^

The memory formation hypothesis is based on associative learning, the association of colony odors with experiences that individuals have with members of these colonies (e.g., attacks, grooming, etc.). This type of learning easily explains the variation in recognition templates and recognition skills observed across workers.^20^^,^^25^^,^^26^^,^^27^^,^^28^

The theory behind the peripheral recognition mechanism suggests that individuals cease to react to the nestmate labels that they are constantly exposed to.^24^^,^^29^^,^^30^ They will only react to novel stimuli, which would include non-nestmate labels. There are two mechanisms potentially involved in this process: habituation and sensory adaptation. Habituation is defined as a decrease in the behavioral response to a constant stimulus^31^ and is a simple form of non-associative learning.^32^ Over time, social insects might gradually ignore their own colony odor and become more sensitive to other odors, e.g., those of non-nestmates. A series of experiments showed that the process of habituation appears to necessitate neurophysiological changes such as synapse plasticity, leading neurons to stop transmitting the information e.g., in the antennal lobe.^16^^,^^18^^,^^33^ Sensory adaptation, in contrast, is a modulation of the olfactory receptors (ORs) at the dendritic membrane of the olfactory sensory neurons (OSNs) in response to a prolonged exposure to a new olfactory environment.^34^^,^^35^ ORs that are overstimulated by specific odorants undergo a process of desensitization, resulting in the loss of perception of the odorants.^29^ Conceptually, both habituation and sensory adaptation blind out stimuli that are invariably present, and allow individuals to continuously be able to perceive relevant stimuli (those that still vary) from their environment and to respond appropriately.

In mammals, a molecular mechanism for olfactory optimization at the OR level has been observed. When exposed to odors, mice modulate their OR gene expression.^36^^,^^37^ Similar alterations in mRNA levels were reported in *Drosophila melanogaster*.^37^^,^^38^ In theoretical models, it was shown that a modulation of OR gene expression and an assumed change in the relative proportions of different ORs in the olfactory periphery (“OR profiles”) can optimize the perception of important cues. The strength of OR expression adjustments depends on receptor tuning: The OR profiles should react more strongly to environmental change when receptor tuning is narrow, and less strongly when there is a large total quantity of neurons.^39^ Insect ORs are G-protein-coupled receptors composed of 3 co-receptor units (ORcos) and a single ORx protein that is odorant-specific.^40^^,^^41^ This complex acts as a metabotropic ionotropic receptor.^42^ In ants, the ORx subunits are thought to be narrowly tuned to single or few odorants.^43^

Previous studies on ants suggested that the particular ORs that an individual expresses are optimized for the tasks that individual performs. Foragers seem to express a specific trail pheromone receptor^44^ and their overall OR gene expression profile also differs from that of nurse ants.^44^^,^^45^

Since OR profiles of individuals vary and OR gene expression is known to respond to changes in the olfactory environment, we speculated that dynamic regulation of OR profiles could also be a mechanism for the habituation-type learning of nestmate recognition odors in the olfactory periphery. If individuals produced fewer ORs binding those substances that are typical to the nestmate odor, e.g., by downregulation of OR gene expression, the sensitivity to nestmate odor would decline as it has been suggested to happen in some circumstances.^16^^,^^18^^,^^24^ In turn, more neurons could be equipped with ORs that are tuned to other substances, which would increase the sensitivity to intruders bearing novel odors.

We show, in line with studies on other species,^10^^,^^13^^,^^16^ that prolonged exposure to non-nestmate odor reduces the aggression of *Acromyrmex echinatior* workers toward the non-nestmate label. We also found that OR gene expression is colony-specific and that it reacts dynamically when individuals are exposed to nestmate recognition labels. The odor exposure affected the OR gene expression profiles in a predictable way, and also affected the expression of other genes that might be involved in regulating OR profiles in the antenna. Interestingly, when we exposed different colonies to the same non-nestmate CHC extract, the initial separation between colonies in the OR gene expression profiles disappeared. This indicates that, as their olfactory environments became similar, their OR profiles also became similar, which could indicate that the nestmate recognition template is in part encoded in the OR profiles.

## Results

**Figure 1:**
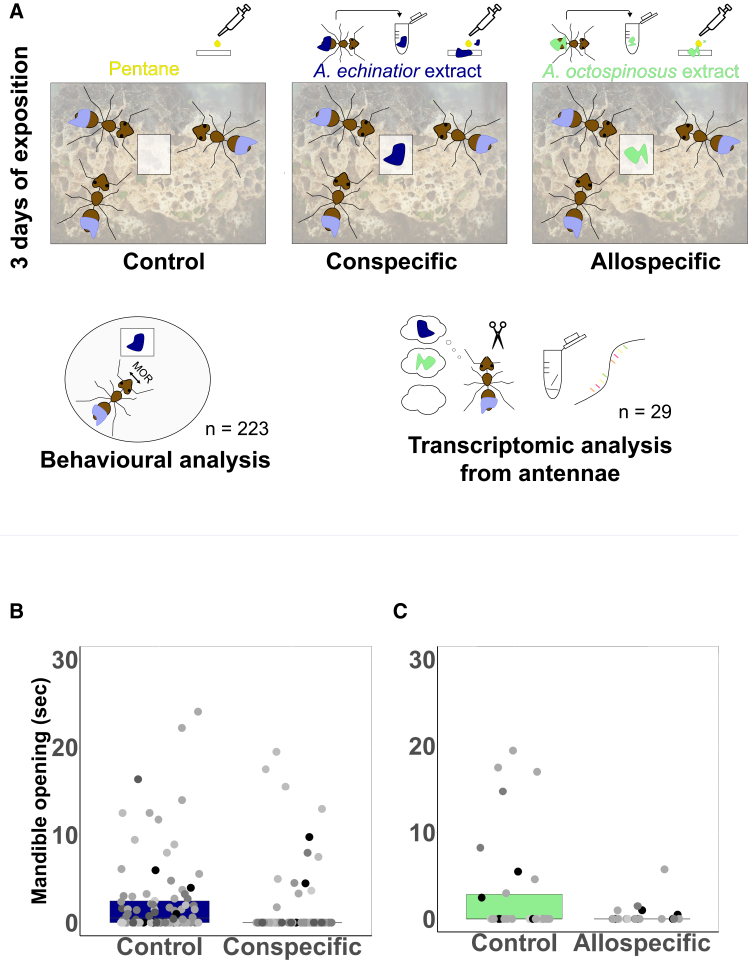
Experimental design and behavioral habituation (A) We established three treatments of odor exposure by setting up 42 subcolonies of 8–10 ants from seven *Acromyrmex echinatior* original colonies and continuously exposed them to either non-nestmate CHC extract from A*cromyrmex echinatior* (conspecific), *Acromyrmex octospinosus* (allospecific), or to solvent (control). On the 4th day, we measured the aggression of the ants (mandible opening duration) and extracted RNA from the antennae for transcriptome analysis. The ants that had been previously exposed to the non-nestmate CHC extract generally reduced their aggressive response toward the same CHC extract compared to the control ants, with a more consistent effect for the allospecific exposure (B, conspecific, *n* = 171, *p* = 0.70, Table S1; C, allospecific, *n* = 52, *p* < 0.01; Table S2). For the behavioral experiment, twelve different combinations of seven focal colonies and eight non-nestmate colonies were used. Each dot is the measurement of an individual ants’ aggression score, the shades correspond to the colony origin of the focal ants. The boxplots indicate medians and interquartile ranges.

Ants are known to incorporate novel odors into their nestmate recognition templates and to habituate to non-nestmate odors during constant exposure.^10^^,^^13^^,^^18^ We explored this effect in *Acromyrmex echinatior* leaf-cutting ants by exposing workers to non-nestmate CHC extracts in experimental subcolonies for three days (Figure 1A). We set up three odor exposure treatments: the control, in which the ants were only exposed to the solvent (pentane); the conspecific exposure, where the ants were exposed to CHC extract of non-nestmates of the same species; and the allospecific exposure, where ants were exposed to the CHCs of another leaf-cutting ant species, *Acromyrmex octospinosus*. We then measured the duration of mandible opening exhibited by ants toward the CHC extract they had been exposed to, and compared it to the control ants’ responses toward the same CHC extract (Figure 1A). When focal ants had previously experienced the allospecific non-nestmate CHC extract, they significantly reduced their aggression toward that odor compared to the control group (Figure 1C, *n* = 56, F_1,51_ = 11.93, *p* < 0.01; Table S2; Figure S2; medians/interquartile ranges for control 0.5 s/0–2.8 s; after exposure 0 s/0–0 s). While there was a similar pattern in the conspecific non-nestmate treatment (Figure 1B), the results were not as conclusive (*n* = 176, F_1,168_ = 0.15, *p* = 0.70; control 0.5 s/0–2.5 s; after treatment: 0 s/0–0 s) because the effect depended on the colony origin of the focal ants (treatment × colony interaction *p* < 0.05; Table S1; Figure S1). Combined with results from previous studies^13^^,^^16^^,^^18^^,^^46^ this evidence suggests that the nestmate recognition template changes toward the non-nestmate cues after exposure to this odor.

Some models predict that the nestmate recognition template may already be encoded at the peripheral level in the antenna.^24^ Since it has been suggested that dynamic OR gene expression might optimize the perception of important cues in vertebrates,^36^^,^^39^ we suspected that the production and hence the overall profile of ORs itself might play a role in template formation in ants as well. We therefore predicted that the relative expression pattern of OR genes may change when the recognition template changes, contributing to the habituation effects observed in nestmate recognition behavior. To test this hypothesis, we analyzed the antennal gene expression of ants from two colonies that we exposed to the same non-nestmate CHC extracts (con- and allo-specific) or to solvent control, as in the behavioral experiment (Figure 1A).

**Figure 2:**
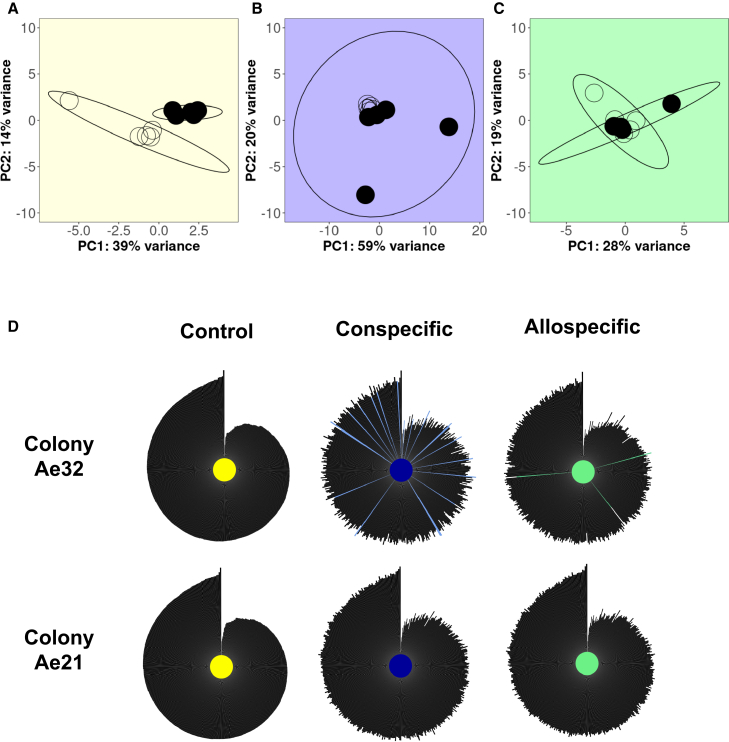
Colony identity and odor exposure affect antennal gene expression OR gene expression differed between the two colonies in the control group (A; *n* = 10, MANOVA *p* < 0.001). This difference disappeared once both colonies were exposed to the same non-nestmate CHC extract, either from a conspecific colony (B; *n* = 10, *p* = 0.17) or an allospecific colony (C; *n* = 9, *p* = 0.78). Each dot represents an individual ants’ OR gene expression profile and the ellipses show 95% confidence levels for each colony. Filled and empty dots indicate the two different colony origins of the focal ants. Exposure to CHC extracts from conspecific (blue) or allospecific (green) non-nestmates modulated the OR profiles in the focal ants’ antennae (D). Each of the 434 bars displayed around the color dots indicates the average relative expression level of one OR gene. In all three subfigures, bars are displayed in the same order, which is sorted according to their expression levels in the control (left/yellow). Bars for OR genes that were differentially expressed compared to the control (DESeq2 p_adj_ < 0.05) are highlighted in color. The smooth shape of the control’s OR profile becomes frayed in the treated groups, indicating that the overall profile changed.

The expression pattern of OR genes in the antennae was colony-specific in the control treatment (Figure 2A, *n* = 10, Manova: Pillai’s Trace = 0.95, F_2,6_ = 66.2, *p* < 0.001). This is expected when OR repertoires are optimized for the perception of important substances, and hence for nestmate recognition. When we exposed both colonies to the same non-nestmate colony CHC extract, the separation between colonies in the OR gene expression disappeared (conspecific, Figure 2B, *n* = 10, Pillai’s Trace = 0.40, F_2,7_ = 2.3, *p* = 0.17; allospecific; Figure 2C, *n* = 9, Pillai’s Trace = 0.10, F_2,6_ = 1.18, *p* = 0.78). This was confirmed in a DESeq2 analysis where 52 out of 435 OR genes were differentially expressed (*p*_adj_ < 0.05) between control ants of the two colonies (Table S5), but this number was significantly reduced to six and seven OR genes after the exposure to con- and allo-specific non-nestmate CHC extract, respectively (Table S5; Pearson’s χ^2^-tests on differentially vs. non-differentially expressed genes between the three treatments, χ^2^ = 67.06, df = 2, *p* < 0.001). Such a pattern indicates that the changes in OR gene expression are specific to the odorants the ants were exposed to. When ants habituate to the CHC extracts of another colony, which effectively changes the nestmate recognition template, OR gene expression adjustments may optimize the sensitivity of the antennae to improve perception of the most relevant cues and accelerate the discrimination of nestmates from non-nestmates. Because the expression of OR genes was colony-specific, we analyzed separately for each experimental colony which ORs were affected by each odor treatment as compared to the control. The exposure to conspecific non-nestmate odor affected the expression of 20 OR genes in colony Ae32, and allospecific odor altered that of three other genes (*p*_adj_ < 0.05, Figure 2D; Table S6). In colony Ae21, no OR genes were differentially expressed after correcting for multiple testing. Interestingly, Ae32 was also the focal colony for which exposure to non-nestmate odor reduced aggression most (Figures S1 and S2). However, the overall profile of OR gene expression always changed, as can be inferred from the frayed shape of the gene expression histograms in Figure 2D. OR profile optimization might thus not be a matter of strong changes in single ORs but rather of small changes in many.

To explore the molecular processes underlying the dynamics of OR gene expression, we analyzed the effect of odor exposure on the expression of all genes. Since we were interested in the genes involved in OR profile dynamics independent of colony identity and the specific odor the ants were exposed to, we accounted for colony ID before testing for the overall treatment effect. Out of the 3,378 genes expressed in the antennae, eight were consistently affected by odor exposure across both colonies (log ratio test *p*_adj_ < 0.05, Table S7). Among these genes we found orthologs of *histone* *human serum albumin* variant (Aech_g03759_i1/NP_001262997.1), three genes with products involved in regulating transcription and splicing (*CWC15 homolog*, *nucleolar protein at 60B*, *mediator complex subunit 21*), and genes encoding two proteins involved in neural development (*tubulin-folding cofactor B*, *tomosyn isoform K*).

**Figure 3:**
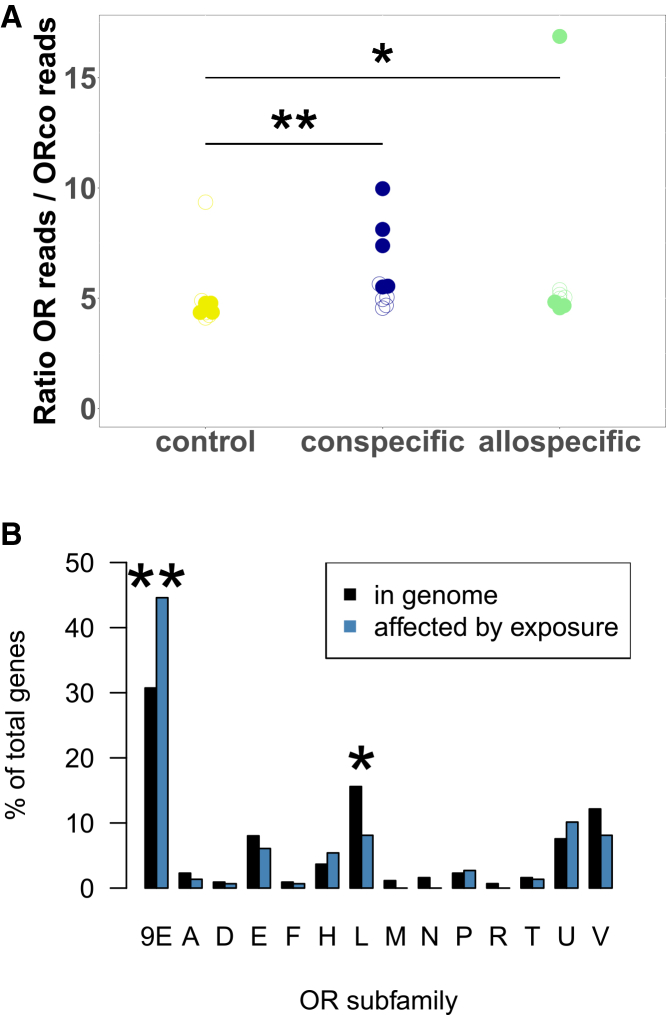
Patterns in the OR gene expression dynamics Exposing ants to non-nestmate odors increased the ratio of OR to *ORco* expression (A). The ratio of the sum of all OR reads in a sample relative to the number of *ORco* reads in the same sample was significantly lower in the control group compared to con- and allospecific exposure (Wilcoxon tests, ∗*p* < 0.05, ∗∗*p* < 0.01). The effect of the allospecific treatment is not only caused by the outlier, but the remaining samples also have generally higher ratios in the allospecific treatment than in the control (*p* = 0.015 after removing one outlier in control and allospecific treatment each). More OR genes of the 9-exon (9E) subfamily were affected by the odor exposure than would be predicted by their proportion among all OR genes in the genome (B; ∗∗ Fisher’s test *p* < 0.01). The L subfamily was underrepresented (∗*p* = 0.02). The list of genes that were affected consisted of both those affected by conspecific and allospecific non-nestmate odor before accounting for the false discovery rate. Only subfamilies containing more than two genes are displayed (Table S8).

To better understand how OR gene expression might relate to the actual receptor profiles in the OSN cell membranes, we investigated the relative expression of the genes coding for tuned ORx receptor components relative to that of the ORco. Each functional receptor complex consists of a tuned Orx receptor and three identical odorant coreceptor subunits,^41^^,^^47^ which predicts that their expression levels should be correlated should all subunits be replaced at the same rate. There was indeed a positive correlation between the number of *ORco* reads and the sum of all other *OR* gene reads (*p* < 0.001, Figure S3; Table S3). However, in our data the total number of all *ORx* reads combined was higher than the number of *ORco* reads (Figure 3A), indicating that some Orx transcripts may not be translated into proteins ending up in a receptor complex, or that tuned and coreceptor subunits are replaced at different rates. Interestingly, the slope of the correlation between the number of *ORco* reads and the total *OR* read count differed between treatments (interaction *ORco* reads: treatment *p* < 0.01, SI Table S3): the experimental odor exposure affected the ratio of *OR* to *ORco* gene expression (Figure 3A), with higher *OR/ORco* ratios found in the ants exposed to the non-nestmate CHC extracts than in pentane (*n* = 29, Wilcoxon test control vs. conspecific non-nestmate odor *p* < 0.01; control vs. allospecific *p* < 0.05). This was not caused by a change in *ORco* expression (Table S6) and should therefore be interpreted as an increase in ORx subunit production after the odor exposure.

Finally, we tested whether some OR subfamilies were affected more by the odor exposure than others. While we did not find evidence for any OR subfamily being enriched among the differentially expressed genes after false discovery rate adjustment (Table S8), we also tested for enrichment using all genes with an initial effect before correction because our previous data indicated that odor exposure had rather small effects on many genes. Intriguingly, more genes of the 9-exon subfamily were affected by the odor exposure than would be expected by chance (Figure 3B; Table S8). In contrast, fewer genes than expected were affected in the L subfamily (Figure 3B; Table S8), although correcting for testing each subfamily separately would render the effect on the L subfamily non-significant (Table S8).

## Discussion

The hydrocarbon mixtures covering the cuticles of social insects are colony-specific and form labels that are stored as neural templates in the memory, allowing nestmate recognition. We show that the gene expression patterns of the ORs that leaf-cutting ants use for the perception of the labels are also colony-specific. This could mean that the ants produce in particular those ORs that help them recognize their nestmates. When ants are exposed to non-nestmate labels, they habituate in a way that reduces aggression against the non-nestmate labels.^16^^,^^18^ We show that at the same time, the OR gene expression profiles in the antennae of the treated ants change. This change was odor-specific, as the OR profiles of two colonies exposed to the same non-nestmate colony converged, indicating that the OR profiles change dynamically according to the current olfactory environment. We found indication that the 9-exon OR subfamily was affected the most, which is fitting given that this subfamily has long been suspected to contain mostly receptors tuned to hydrocarbons, the primary nestmate recognition substances.^6^^,^^43^^,^^48^^,^^49^

Our findings open up the possibility that the antennal OR profiles are optimized for nestmate recognition. To discriminate nestmates from non-nestmates, certain substances are likely more useful than others: substances that vary randomly, as well as those found on nestmates and non-nestmates in similar quantities, are not very informative. Their perception is thus irrelevant for nestmate recognition. In contrast, other substances may differ strongly between friends and enemies, and increasing the sensitivity to those substances would improve nestmate recognition. As has been hypothesized before, changes in OR expression could alter the receptor profiles across the antenna and could optimize recognition.^39^

One specific case of this optimization is described as the pre-filter mechanism for nestmate recognition. This hypothesis suggests that neurons that are constantly activated by nestmate-specific substances become desensitized, resulting in the loss of perception of these odorants.^24^ This sensory adaptation should enable individuals to better perceive potential non-nestmate stimuli appearing in their environment. If the production of ORs tuned to nest-specific substances was reduced, this would result in a lowered sensitivity (and lowered reaction to) nest-specific substances. In turn, this downregulation could potentially free resources for increasing the numbers of ORs tuned to substances best suited to perceive novel odors and thus intruders. Typically, desensitization is expected to occur to receptor complexes^50^ or to neural networks (e.g., in the antennal lobe), but changes in OR numbers across the antenna would have similar effects.

An alternative explanation for our findings would be that the change in OR gene expression is a more or less passive response to OR breakdown caused by OR use. It is plausible that odorant binding to receptors causes wear-and-tear to the receptor complexes. If this causes breakdown of receptor parts, the cell might repair receptors by replacing the entire receptor complex or its parts.^37^^,^^38^ In our experiment, *ORco* expression remained constant, so that it is unlikely that the entire receptor complexes were replaced. While the explanation that ORx subunits are replaced may be parsimonious, some of our observations suggest that there are indeed active changes that would be expected if the OR profiles were constantly optimized for recognition. These clues come from the experimental change of the recognition templates by exposure to non-nestmate labels. Odor exposure did not only trigger changes in OR expression as would be predicted by a passive tracking of damage caused by the most common odorants at any given time. Instead, the exposure also triggered changes in gene expression in the antenna that could indicate that the receptor profiles are actively modified independent of wear and tear of OR complexes. The tubulin-binding cofactor B (ortholog of *Aech_g10112_i1*), for example, is involved in the regulation of neurogenesis in mammals,^51^ and tomosyn (*Aech_g14430_i1*, syntaxin-binding protein 5) is involved in regulating synaptic vesicles so that its knockdown can affect memory formation in *Drosophila*.^52^ We also find genes differentially expressed that are involved in the regulation of transcription or processing of RNA (e.g., splicing) (*Aech_g12595_i1*: mediator complex subunit 21, *Aech_g15049_i1*: nucleolar protein at 60B, and *Aech_g03759_i1*: spliceosome-associated protein CWC15 homolog). A particularly interesting gene is *histone H2A*.*V* (*Aech_g03759_i1*). Its expression is affected by odor exposure as well, hinting at the possibility that histone modification may be a mechanism to regulate colony-specific OR profiles.^53^

How exactly the OR profiles are altered is still unclear. Usually, each OSN possesses only one ORx type in the membrane, i.e., one OSN is responsible for the perception of one odorant. OR profile alterations could thus happen through increasing the density of important ORs on the membranes of their respective OSNs, or by replacing the OSNs altogether, which occurs regularly in ants, *Drosophila*, and mammals.^54^^,^^55^^,^^56^ In our experiment, the *OR*:*ORco* gene expression ratio systematically increased after we changed the recognition templates. OR complexes are typically made up by three identical odorant coreceptor proteins and one odorant receptor.^41^^,^^47^ In our control ants, the *OR*:*ORco* gene expression ratio was around five, but this ratio increased in particular in ants exposed to conspecific non-nestmate odor—indicating that more ORx subunits were produced when the label changed. Since the ORx are the receptor components that are specifically tuned to odorants, one would indeed expect them to be more affected by specific odorant changes than the generic co-receptor ORco, should only the odorant-specific receptor subunits be swapped. In contrast, the most parsimonious expectation for neuron turnover would be that ORco and ORx expression would be equally affected. It is thus tempting to speculate that neurons might replace the ORx subunits of the receptor. This would suggest that in case of a systematic change in the olfactory environment, some OR subunits might be swapped out to optimize the sensitivity to important cues, while the co-receptor subunits would be retained.

No matter the mechanism, we show that OR gene expression in ants is colony-specific and dynamically adapts to a changing nestmate template. It is tempting to speculate that the flexibility of OR profiles contributes an optimization of nestmate recognition, indicating that template formation may start in the antenna, possibly as part of a pre-filter mechanism.^24^ Earlier studies have suggested the involvement of higher brain centers in the formation of the nestmate recognition template.^21^ These processes are by no means mutually exclusive. We suggest that a pre-filter mechanism could act as a secondary mechanism in parallel with learning, or could even be activated by learning. When specific labels are important enough to be learned, it would be beneficial to be able to accurately identify them. Learning might thus trigger feedback to the antennal lobe and the OSNs to optimize the perception of and discrimination among relevant labels. The nestmate recognition template can be seen not as a single neural representation located in a specific compartment of the neural system, but rather a complex of neural processes from the periphery up to the central nervous system.^57^ The sum of modulations that occur in parallel in central and peripheral olfactory systems could work in synergy and optimize nestmate recognition. This view reconciles different lines of evidence for both peripheral and central nervous system templates.^18^^,^^20^

### Limitations of the study

The RNA-seq dataset in this study consists of samples from workers of only two colonies. While these two colonies clearly differ in their OR gene expression before odor exposure, it is possible that the magnitude of the differences between colonies vary when considering different pairs of colonies. We already know that there is quite some variation in recognition between colony pairs,^20^ and this is also visible in our behavioral dataset. RNA-seq data from more colonies, combined with behavioral data involving the same colonies, would help us to better understand this type of variation.

The discussion of potential mechanisms leading to colony-specific and odor-responsive OR gene expression profiles is largely based on correlations in the expression of different genes in our RNA-seq dataset. They remain speculative and would benefit from functional studies.

## Resource availability

### Lead contact

Further information and requests for resources and reagents should be directed to and will be fulfilled by the lead contact, Volker Nehring (volker.nehring@biologie.uni-freiburg.de).

### Materials availability

This study did not generate new unique reagents.

### Data and code availability

• Raw behavioral data are available from Table S9. Raw sequences are available from NCBI under the BioProject: PRJNA1147124.
• All original code is available in File S1 of the manuscript.
• Any additional information required to reanalyze the data reported in this study is available from the lead contact upon request.

## Acknowledgments

We would like to thank the Department of Ecology & Evolution for stimulating discussions and the Global Ant Genomics Alliance for providing us with early access to the chromosome-level *Acromyrmex echinatior* genome. We would also like to thank the 10.13039/501100001659German Research Foundation (NE 1969/6-1) for funding. Our thanks also go to the Smithsonian Tropical Research Institute in Panama for providing access to their laboratory facilities and to the Autoridad Nacional del Ambiente of Panama for issuing collection and export permits.

## Author contributions

M.B., N.J.L.A., and L.M. conducted the behavioral experiments. M.B. conducted the molecular laboratory work and analysis, to which Y.P. and J.V. contributed the OR subfamily categorization. M.B. and V.N. wrote the manuscript with input from J.V.

## Declaration of interests

The authors declare no competing interests.

## STAR★Methods

### Key resources table

**Table:** 

REAGENT or RESOURCE	SOURCE	IDENTIFIER
**Deposited data**		

Raw RNA sequences	This paper	PRJNA1147124
Acromyrmex echinatior genome	Vizueta et al., 2025^58^	GAGA-0014

**Software and algorithms**		

Boris version 7.10.55	Friard and Gamba, 2016^59^	
R 4.0.5	R Development Core Team, 2016^60^	
fastp	Chen et al., 2018^61^	
MultiQC	Ewels et al., 2016^62^	
HISAT2	Kim et al., 2019^63^	
SAMtools	Danecek et al., 2021^64^	
HTSeq	Anders et al., 2015^65^	
DESeq2	Love et al., 2014^66^	

### Experimental model and study participant details

Twelve queenright *Acromyrmex* colonies collected in Gamboa, Panama between 2017 and 2022 were used for behavioural experiments (9 colonies of *Acromyrmex echinatior*, 3 colonies of *Acromyrmex octospinosus*). Two of the *A*. *echinatior* colonies were also used for transcriptomic analysis. The ants were maintained at the University of Freiburg at a temperature of 25 – 28°C and 70 - 80% humidity with a 12 h light–dark photoperiod in fluon-coated plastic boxes (from 39 x 28 x 14 up to 57 x 39 x 28 cm, depending on the colony size). Each box contained fungus gardens grown in beakers (from 0.5 up to 1L, according to the colony size) covered by upturned plastic flowerpots. The colonies were provided with bramble, rice, and apple slices, and sprayed with water twice a week. The experiments were conducted from December 2021 to February 2023.

### Method details

#### Experimental design

We tested whether an *Acromyrmex echinatior* ant constantly exposed to a new cuticular hydrocarbon profile extract would modulate its behaviour toward that CHC extract, and whether the exposure changed the gene expression in its antennae. We exposed ants in subcolonies to CHC extracts from different colonies belonging to the same or a closely related species. Afterwards, we measured the aggression toward the non-nestmate CHC extract, and the gene expression in the antennae (Figure 1A). From 7 *Acromyrmex echinatior* original colonies, we set up 42 subcolonies that we exposed to three different exposure treatments. For each subcolony, 8 to 10 medium-sized forager ants were set up in fluon-coated© plastic boxes (10 x 10 cm) and provided with fungus, bramble leaves, and moist cotton (Table S4). We used foragers because these tend to be more aggressive than nurses (e.g., Larsen et al. 2016), a pattern that we have also observed in *A*. *echinatior* (unpublished data). On the following day, we began to expose each subcolony to either solvent control, conspecific non-nestmate odour, or allospecific non-nestmate odour, for three days. We used glass slides covered with either pentane solvent (control), non-nestmate CHC extract from *A*. *octospinosus* (allospecific) or from *A*. *echinatior* (conspecific), and replaced the slides once a day (Figure 1A). After three days of exposure (a period longer than needed for successful habituation in other ant species, e.g. Guerrieri et al. 2009), we tested whether the ants previously exposed to non-nestmate CHC extract would decrease their aggression towards the CHC extract they had been exposed to. Half of the control ants were tested for their reaction to conspecific non-nestmate CHC extract, and the other half to allospecific CHC extract. For two *A*. *echinatior* colonies, some of the ants were not used for the behavioural experiment but for the transcriptomic analysis instead (Table S4). Their antennae were dissected and the RNA extracted separately for each individual. RNA was sent for sequencing for 28 ants in total.

#### CHC extracts

Medium size foraging workers were killed by freezing at -20°C for 45 minutes, then placed in a glass beaker and covered with n-pentane for three minutes on an orbital shaker (Phero Shaker, Mod.:13A34, Biotec Fischer GmbH) to extract the cuticular hydrocarbons (CHCs) from the ants’ cuticles. The ants were removed using a metal sieve and the solvent was evaporated. Then, pentane was added to achieve a concentration of 1 ant per 11 μL. The extracts were kept in 5ml glass tubes that were renamed with codes to ensure blind experimentation. We applied 22 μL (2 ant equivalents) of the solution onto glass slides and let them dry before placing them into the subcolonies for the experimental CHC exposure.

#### Behavioural analysis

After 3 days of odour exposure, on day 4, we tested whether the ants would react aggressively to the odour or to solvent control. Behavioural assays were conducted in a circular fluon-coated arena placed on filter paper (Ø 5 cm). A dried slide of 11 μL (1 ant equivalent) of CHC extract was placed inside a smaller plastic cylinder (Ø 2.5 cm) in the middle of the arena and the focal ant was placed outside this cylinder to prevent contact. The ants were left to acclimatize to the arena for five minutes before the small cylinder was removed to allow contact between the individuals and the slide. Tests were live- or video-recorded. The focal ants’ behaviours were quantified for three minutes using the software Boris (version 7.10.55^59^ and the observer was always blind since both the focal ant’s previous exposure and the origin of the CHC extract for the test were unknown. Mandible opening duration near the slide was recorded. Mandible opening is a precursor of biting behaviour and a good estimate of aggression.^67^ We had conducted preliminary tests before the experiment to ensure that the focal colonies were aggressive against the colonies used to obtain the CHC extract.

#### Transcriptome analysis

Antennal tissues were dissected after 3 days of exposure, on day 4. Each sample was composed of both antennas from a single individual. Just after dissection, the antennae were placed in 50 μl peqGOLD Tri Fast™ and crushed with 2 Zirconia beads in a Tissue Lyser II, for 2 minutes at 25 Hz, and where then preserved at -80°C until extraction. RNA was extracted from the antenna following an optimised RNA isolation protocol using a Qiagen kit, RNeasy©. First, we thawed the tissue for 2 to 3 min and after adding 200 μl peqGOLD Tri Fast™, and homogenized the sample. Then, we added 50 μl of chloroform to separate the aqueous phase, before 100 μl isopropanol was added to precipitate the RNA. After the content was transferred into an RNeasy Mini spin column, we washed the column with the RPE buffer provided by the kit, which we had mixed with ethanol. The sample was centrifuged for 1 min at 4°C, 11000 xg. The washing step was repeated three times. Finally, the RNA attached to the column membrane was dissolved in 30 μl nuclease-free water.

The RNA samples were kept at -80°C until they were sent to BGI, Hong Kong. The libraries were prepared by BGI as DNBSEQ Low Input Smart-Seq Eukaryotic mRNA library. Amplified libraries were sequenced on the DNBseq platform (100bp paired-end reads), generating ∼8 Gigabases of raw data for each sample.

RNASeq raw reads were trimmed using fastp^61^ and filtered using MultiQC.^62^ The remaining sequences were mapped to the *Acromyrmex echinatior* chromosome-level genome^58^ using HISAT2^63^ with default parameters. The SAM files generated were converted to BAM files and sorted by name using SAMtools,^64^ which were used to generate gene count tables using HTSeq^65^ with the following settings: -f bam, -i transcript_id, -t CDS, -m union, -r name, --stranded=no.

### Quantification and statistical analysis

#### Behavior

Some ants never contacted the slide covered with colony odours; we removed these data points from the dataset, leaving 232 observations in total (Table S4). The duration of mandible opening behaviour (continuous variable from 0 to ca. 36 seconds, roughly following a Poisson-distribution) was set up as the dependent variable in generalized linear models (GLM) with quasi-poisson error family due to overdispersion (dispersion parameter > 5 in all cases). The different exposure (allospecific or conspecific non-nestmate vs. control), the colony origin of focal ants, and their interaction were used as predictors. The experiments with allo- and conspecific non-nestmate CHC extracts were analysed separately. Significance testing was done using variance analysis tests (anova.glm() function of the stats library) after checking residual plots for heteroscedasticity. The results were interpreted as significant when *p* < 0.05 (two-tailed). All statistical analyses were performed using R 4.0.5 software.^60^

#### Transcriptome data

We determined the differentially expressed genes (DEGs) between workers exposed to CHCs and pentane (control) using the generalized negative binomial model implemented in DESeq2.^66^ P-values were calculated by Wald tests and corrected for multiple testing using the false discovery rate approach. Genes with adjusted *p*-values p_adj_ < 0.05 were listed as DEGs. To visualize the results, we normalized the gene count data using the rlogTransformation function from DESeq2, which transforms the count data to the log_2_ scale in a way which minimizes differences between samples for rows with small counts. We performed a principal component analysis (PCA) with the plotPCA function from the DESeq2 package. All statistical analyses were performed using R 4.0.5 software.^60^ We used blastn to find *Drosophila* orthologues of non-OR genes of interest. We tested whether any OR subfamily (based on the categorization from Pellen et al.^49^) was particularly affected by our experimental treatment (Table S8). To do so, we created a list of OR genes containing all genes that were either affected by the allospecific or by the conspecific non-nestmate treatment as compared to control. Because we suspected that small effects on many genes play a role in the context we investigated, we tested not only OR genes with p_adj_ < 0.05, but also a list of all OR genes with an uncorrected *p* < 0.05. We compared for each OR family separately whether it was more or less affected than would be expected based on the number of genes it contains using Fisher’s exact tests.

## Supplemental information

Document S1. Figures S1–S3 and Tables S1–S4 and S8

Table S5. DESeq2 analysis results for OR comparison between colonies, separately per treatment, related to Figures 2A–2C

Table S6. Treatment effect on OR expression, separately per colony, related to Figure 2D

Table S7. DESEQ2 results for the exposure effect on all expressed genes, including blast hits for significant DEGs, related to Figure 2

Table S9. Raw data from the behavioral experiment, related to Figure 1

File S1. Read count table and code for transcriptome analysis, related to Figures 2 and 3

## References

[1] Tan Y., Li W.H. (1999). Trichromatic vision in prosimians. Nature.

[2] Marasco P.D., Catania K.C. (2007). Response properties of primary afferents supplying Eimer’s organ. J. Exp. Biol..

[3] Wyatt T.D. (2014).

[4] Benton R. (2022). *Drosophila* olfaction: past, present and future. Proc. Biol. Sci..

[5] Lenoir A., Fresneau D., Errard C., Hefetz A., Detrain C., Deneubourg J.L., Pasteels J.M. (1999). Information Processing in Social Insects.

[6] Leonhardt S.D., Menzel F., Nehring V., Schmitt T. (2016). Ecology and evolution of communication in social insects. Cell.

[7] Sturgis S.J., Gordon D.M. (2012). Nestmate recognition in ants (Hymenoptera: Formicidae): a review. Myrmecological News.

[8] van Zweden J.S., d’Ettorre P., Blomquist G.J., Bagneres A.G. (2010). Insect Hydrocarbons: Biology, Biochemistry, and Chemical Ecology.

[9] Sherman P.W., Reeve H.K., Pfennig D.W., Krebs J.R., Davies N.B. (1997). Behavioural Ecology.

[10] Errard C., Hefetz A. (1997). Label familiarity and discriminatory ability of ants reared in mixed groups. Insectes Soc..

[11] Le Moli F., Mori A. (1990). Laboratory experiments on environmental sources of nestmate and non-nestmate discrimination in three species of formica ants (Hymenoptera: Formicidae). Psyche: J. Entomol..

[12] Leonhardt S.D., Brandstaetter A.S., Kleineidam C.J. (2007). Reformation process of the neuronal template for nestmate-recognition cues in the carpenter ant *Camponotus floridanus*. J. Comp. Physiol..

[13] Carlin N.F., Hölldobler B. (1983). Nestmate and kin recognition in interspecific mixed colonies of ants. Science.

[14] Dahbi A., Cerdá X., Hefetz A., Lenoir A. (1996). Social closure, aggressive behavior, and cuticular hydrocarbon profiles in the polydomous ant *Cataglyphis iberica* (Hymenoptera, Formicidae). J. Chem. Ecol..

[15] Foubert E., Nowbahari E. (2008). Memory span for heterospecific individuals’ odors in an ant, Cataglyphis cursor. Learn. Behav..

[16] Guerrieri F.J., Nehring V., Jørgensen C.G., Nielsen J., Galizia C.G., d’Ettorre P. (2009). Ants recognize foes and not friends. Proc. Biol. Sci..

[17] Neupert S., Hornung M., Grenwille Millar J., Kleineidam C.J. (2018). Learning distinct chemical labels of nestmates in ants. Front. Behav. Neurosci..

[18] Stroeymeyt N., Guerrieri F.J., van Zweden J.S., d’Ettorre P. (2010). Rapid decision-making with side-specific perceptual discrimination in ants. PLoS One.

[19] Dahbi A., Hefetz A., Cerdá X., Lenoir A. (1999). Trophallaxis mediates uniformity of colony odor in Cataglyphis iberica ants (Hymenoptera, Formicidae). J. Insect Behav..

[20] Bey M., Endermann R., Raudies C., Steinle J., Nehring V. (2025). Associative learning of non-nestmate cues improves enemy recognition in ants. Curr. Biol..

[21] Bos N., d’Ettorre P. (2012). Recognition of social identity in ants. Front. Psychol..

[22] Brandstaetter A.S., Rössler W., Kleineidam C.J. (2011). Friends and foes from an ant brain’s point of view – neuronal correlates of colony odors in a social insect. PLoS One.

[23] Nehring V., Dani F.R., Calamai L., Turillazzi S., Bohn H., Klass K.D., D’Ettorre P. (2016). Chemical disguise of myrmecophilous cockroaches and its implications for understanding nestmate recognition mechanisms in leaf-cutting ants. BMC Ecol..

[24] Ozaki M., Hefetz A. (2014). Neural mechanisms and information processing in recognition systems. Insects.

[25] Esponda F., Gordon D.M. (2015). Distributed nestmate recognition in ants. Proc. Biol. Sci..

[26] Larsen J., Nehring V., d’Ettorre P., Bos N. (2016). Task specialization influences nestmate recognition ability in ants. Behav. Ecol. Sociobiol..

[27] Larsen J., Fouks B., Bos N., D’Ettorre P., Nehring V. (2014). Variation in nestmate recognition ability among polymorphic leaf-cutting ant workers. J. Insect Physiol..

[28] Norman V.C., Hoppé M., Hughes W.O.H. (2014). Old and wise but not size: factors affecting threat response behaviour and nestmate recognition in *Acromyrmex echinatior* leaf-cutting ants. Insectes Soc..

[29] d’Ettorre P., Lenoir A., Lach L., Parr C., Abbott K. (2010). Ant Ecology.

[30] Reeve H.K. (1989). The evolution of conspecific acceptance thresholds. Am. Nat..

[31] Rankin C.H., Abrams T., Barry R.J., Bhatnagar S., Clayton D.F., Colombo J., Coppola G., Geyer M.A., Glanzman D.L., Marsland S. (2009). Habituation revisited: An updated and revised description of the behavioral characteristics of habituation. Neurobiol. Learn. Mem..

[32] Kandel E.R. (2004). The molecular biology of memory storage: a dialog between genes and synapses. Biosci. Rep..

[33] Langen T.A., Tripet F., Nonacs P. (2000). The red and the black: habituation and the dear-enemy phenomenon in two desert *Pheidole* ants. Behav. Ecol. Sociobiol..

[34] Todd J.L., Baker T.C., Hansson B.S. (1999). Insect Olfaction.

[35] Wark B., Lundstrom B.N., Fairhall A. (2007). Sensory adaptation. Curr. Opin. Neurobiol..

[36] Ibarra-Soria X., Nakahara T.S., Lilue J., Jiang Y., Trimmer C., Souza M.A., Netto P.H., Ikegami K., Murphy N.R., Kusma M. (2017). Variation in olfactory neuron repertoires is genetically controlled and environmentally modulated. eLife.

[37] Von Der Weid B., Rossier D., Lindup M., Tuberosa J., Widmer A., Col J.D., Kan C., Carleton A., Rodriguez I. (2015). Large-scale transcriptional profiling of chemosensory neurons identifies receptor-ligand pairs in vivo. Nat. Neurosci..

[38] Koerte S., Keesey I.W., Khallaf M.A., Cortés Llorca L., Grosse-Wilde E., Hansson B.S., Knaden M. (2018). Evaluation of the DREAM technique for a high-throughput deorphanization of chemosensory receptors in *Drosophila*. Front. Mol. Neurosci..

[39] Teşileanu T., Cocco S., Monasson R., Balasubramanian V. (2019). Adaptation of olfactory receptor abundances for efficient coding. eLife.

[40] Suh E., Bohbot J., Zwiebel L.J. (2014). Peripheral olfactory signaling in insects. Curr. Opin. Insect Sci..

[41] Zhao J., Chen A.Q., Ryu J., Del Mármol J. (2024). Structural basis of odor sensing by insect heteromeric odorant receptors. Science.

[42] Gomez-Diaz C., Martin F., Garcia-Fernandez J.M., Alcorta E. (2018). The two main olfactory receptor families in *Drosophila*, ORs and IRs: a comparative approach. Front. Cell. Neurosci..

[43] Pask G.M., Slone J.D., Millar J.G., Das P., Moreira J.A., Zhou X., Bello J., Berger S.L., Bonasio R., Desplan C. (2017). Specialized odorant receptors in social insects that detect cuticular hydrocarbon cues and candidate pheromones. Nat. Commun..

[44] Koch S.I., Groh K., Vogel H., Hansson B.S., Kleineidam C.J., Grosse-Wilde E., Grosse-Wilde E. (2013). Caste-specific expression patterns of immune response and chemosensory related genes in the leaf-cutting ant, *Atta vollenweideri*. PLoS One.

[45] Caminer M.A., Libbrecht R., Majoe M., Ho D.V., Baumann P., Foitzik S. (2023). Task-specific odorant receptor expression in worker antennae indicates that sensory filters regulate division of labor in ants. Commun. Biol..

[46] Couvillon M.J., Caple J.P., Endsor S.L., Kärcher M., Russell T.E., Storey D.E., Ratnieks F.L.W. (2007). Nest-mate recognition template of guard honeybees (Apis mellifera) is modified by wax comb transfer. Biol. Lett..

[47] Yan H. (2025). Insect olfactory neurons: receptors, development and function. Curr. Opin. Insect Sci..

[48] Engsontia P., Sangket U., Robertson H.M., Satasook C. (2015). Diversification of the ant odorant receptor gene family and positive selection on candidate cuticular hydrocarbon receptors. BMC Res. Notes.

[49] Pellen Y., Vizueta J., Beck E., Liebig J., Schrader L., Privman E. (2026). Adaptive evolution of odorant receptors is associated with elaborations of social organization in ants. Mol. Biol. Evol..

[50] Wicher D. (2018). Tuning insect odorant receptors. Front. Cell. Neurosci..

[51] Lopez-Fanarraga M., Carranza G., Bellido J., Kortazar D., Villegas J.C., Zabala J.C. (2007). Tubulin cofactor B plays a role in the neuronal growth cone. J. Neurochem..

[52] Chen K., Richlitzki A., Featherstone D.E., Schwärzel M., Richmond J.E. (2011). Tomosyn-dependent regulation of synaptic transmission is required for a late phase of associative odor memory. Proc. Natl. Acad. Sci. USA.

[53] Baldi S., Becker P.B. (2013). The variant histone H2A.V of *Drosophila*—three roles, two guises. Chromosoma.

[54] Fernández-Hernández I., Hu E., Bonaguidi M.A. (2020). Olfactory neuron turnover in adult Drosophila. bioRxiv.

[55] Lledo P.M., Gheusi G., Vincent J.D. (2005). Information processing in the mammalian olfactory system. Physiol. Rev..

[56] Yan H., Opachaloemphan C., Mancini G., Yang H., Gallitto M., Mlejnek J., Leibholz A., Haight K., Ghaninia M., Huo L. (2017). An engineered orco mutation produces aberrant social behavior and defective neural development in ants. Cell.

[57] Sung J.Y., Harris O.K., Hensley N.M., Chemero A.P., Morehouse N.I. (2021). Beyond cognitive templates: re-examining template metaphors used for animal recognition and navigation. Integr. Comp. Biol..

[58] Vizueta J., Xiong Z., Ding G., Larsen R.S., Ran H., Gao Q., Stiller J., Dai W., Jiang W., Zhao J. (2025). Adaptive radiation and social evolution of the ants. Cell.

[59] Friard O., Gamba M. (2016). BORIS : a free, versatile open-source event-logging software for video/audio coding and live observations. Methods Ecol. Evol..

[60] R Development Core Team (2016).

[61] Chen S., Zhou Y., Chen Y., Gu J. (2018). fastp: an ultra-fast all-in-one FASTQ preprocessor. Bioinformatics.

[62] Ewels P., Magnusson M., Lundin S., Käller M. (2016). MultiQC: summarize analysis results for multiple tools and samples in a single report. Bioinformatics.

[63] Kim D., Paggi J.M., Park C., Bennett C., Salzberg S.L. (2019). Graph-based genome alignment and genotyping with HISAT2 and HISAT-genotype. Nat. Biotechnol..

[64] Danecek P., Bonfield J.K., Liddle J., Marshall J., Ohan V., Pollard M.O., Whitwham A., Keane T., McCarthy S.A., Davies R.M., Li H. (2021). Twelve years of SAMtools and BCFtools. GigaScience.

[65] Anders S., Pyl P.T., Huber W. (2015). HTSeq-A Python framework to work with high-throughput sequencing data. Bioinformatics.

[66] Love M.I., Huber W., Anders S. (2014). Moderated estimation of fold change and dispersion for RNA-seq data with DESeq2. Genome Biol..

[67] Guerrieri F.J., d’Ettorre P. (2008). The mandible opening response: quantifying aggression elicited by chemical cues in ants. J. Exp. Biol..

