# Supplementary material for: Exposure to non-nestmate odors changes the odorant receptor profile in Acromyrmex echinatior leaf-cutting ants
Source: iScience. 2026 Jul 13;29(8):116665. doi: 10.1016/j.isci.2026.116665 (PMC13382326; doi:10.1016/j.isci.2026.116665)
Supplement: Document S1. Figures S1–S3 and Tables S1–S4 and S8 [file mmc1.pdf]

iScience, Volume *29*

Supplemental information

Exposure to non-nestmate odors changes the odorant receptor profile in *Acromyrmex echinatior* leaf-cutting ants

Mélanie Bey, Naomi Jeanne Luna Alex, Lisa Maczkowicz, Yoann Pellen, Joel Vizueta, and Volker Nehring

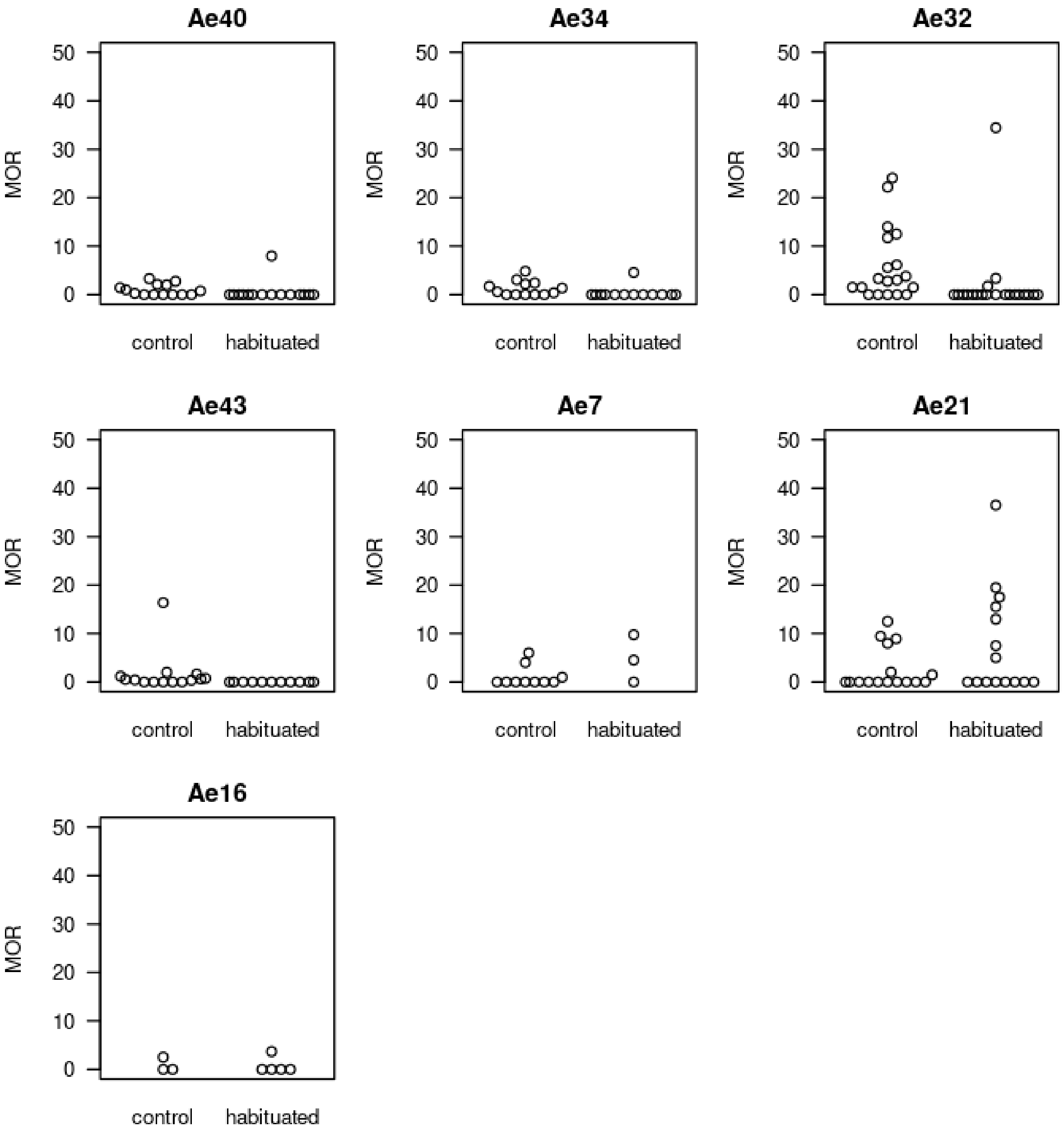

Figure S1: The effects of intraspecific habituation separately for each focal colony. Focal colonies are the 7 colonies of origin the observed ants came from. For colonies Ae32 and Ae21, we also generated antenna transcriptomes. When tested separately, exposing the ants to intraspecific non-nestmate odour reduced the aggression of ants from colony Ae32 (n = 39, U-Test W = 300, $p < 0.001$), but this was not evident for colony Ae21 (n = 31, W = 96, p = 0.30). Related to Fig. 1B.

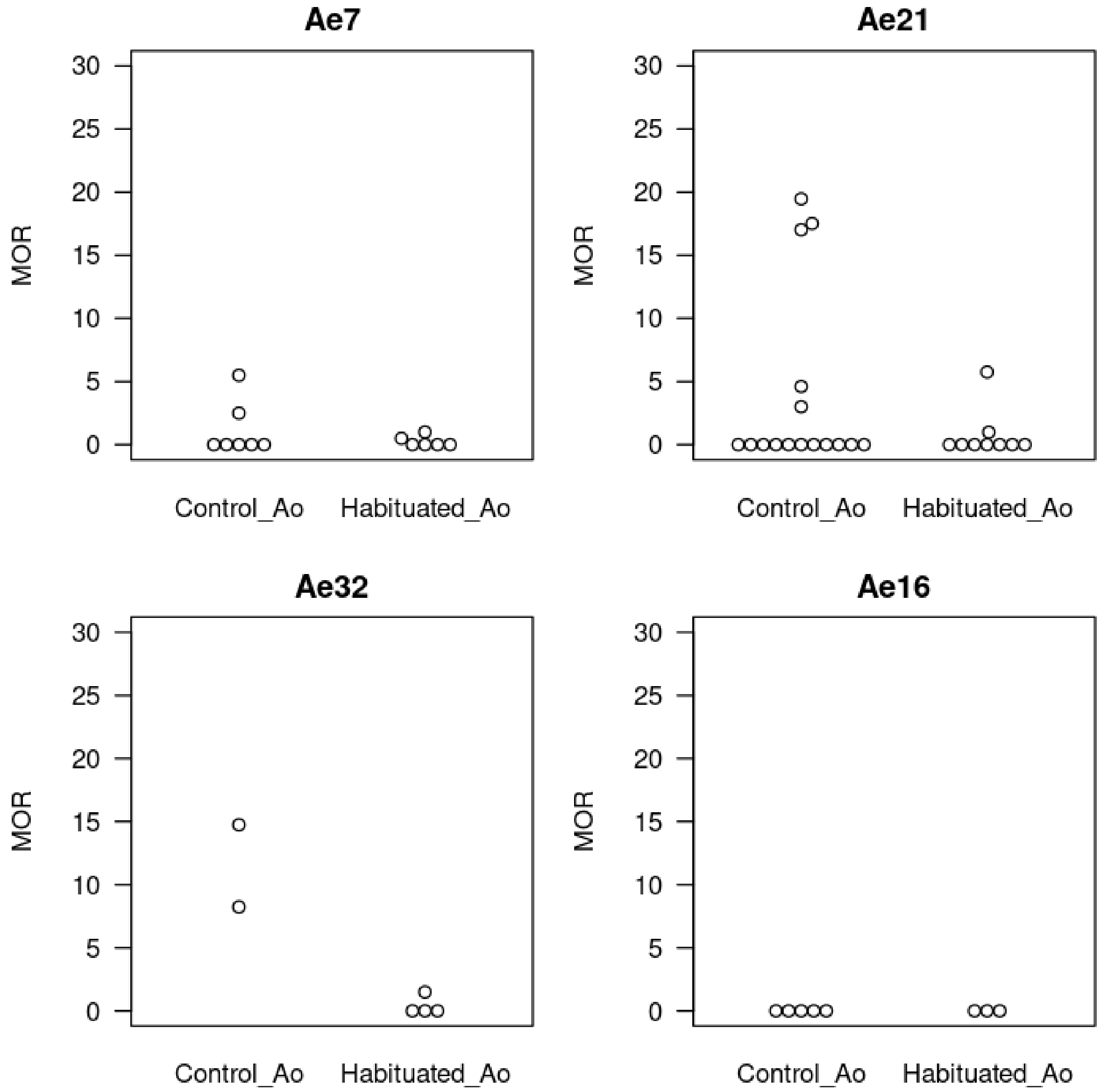

Figure S2: The effects of interspecific habituation separately for each focal colony. Focal colonies are the 4 colonies of origin the observed ants came from. For colonies Ae32 and Ae21, we also generated antenna transcriptomes. When tested separately, there was no clear effect of exposing ants from colony Ae32 to interspecific odour ($n = 6$, U-Test $W = 8$, $p = 0.09$) but the sample size was very low for this colony. For colony Ae21, no effect was evident ($n = 25$, $W = 82$, $p = 0.52$). Related to Fig. 1C.

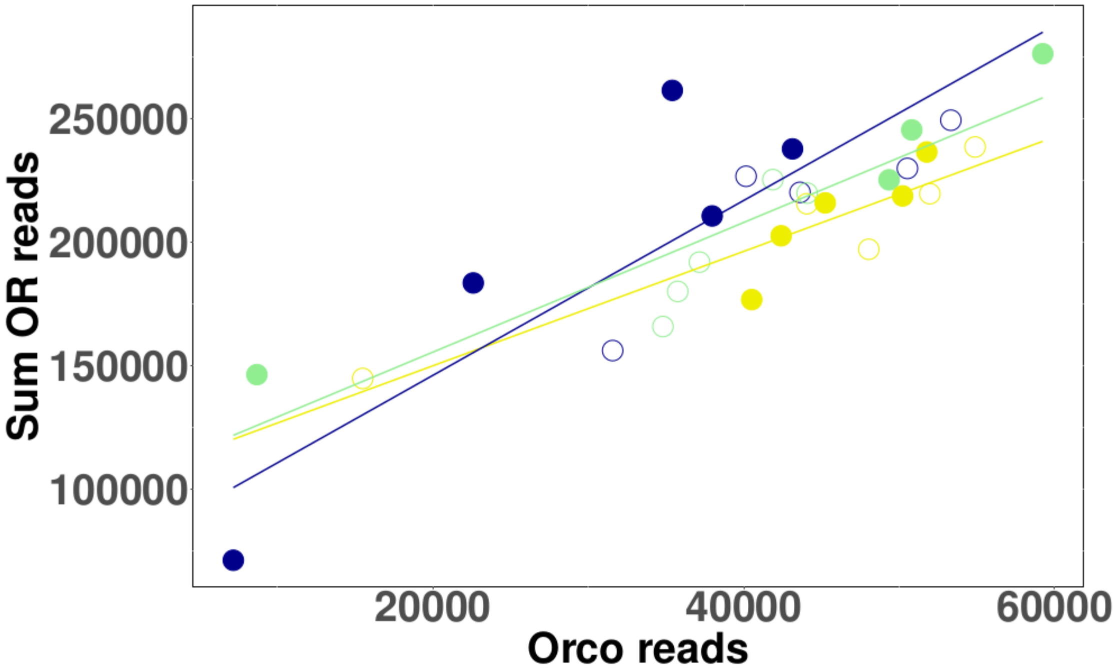

Figure S3: The total OR gene expression was positively correlated with the expression of the OR coreceptor ORco. The sum of the OR gene reads depended on the number of ORco reads (n = 29 individuals, $p < 0.001$, Tab. S3) and the odour ants have been exposed to (n = 8-10 per treatment, $p < 0.05$, Tab. S3). Each dot is the measurement of an individual ant's gene expression, the different colours correspond to the different treatments the ants were exposed to. Filled and empty dots indicate individuals from two different colonies. Related to Fig. 3A.

Table S1: Analysis of Variance on a GLM with quasi-poisson error family, describing the duration of mandible opening in reaction to conspecific non-nestmate CHC extract. Related to Fig. 1.
Full model: mandible opening ~ Colony origin focal ants x Treatment

Colony origin of focal ants: $F_{6,164} = 4.01$, $p = 0.001$
Treatment: $F_{1,163} = 1.37$, $p = 0.702$
Colony origin of focal ants x Treatment: $F_{6,157} = 2.30$, $p = 0.037$

Table S2: Analysis of Variance on a GLM with quasi-poisson error family, describing the duration of mandible opening in reaction to allospecific non-nestmate CHC extract. Related to Fig. 1.
Full model: mandible opening ~ Colony origin focal ants x Treatment

Colony origin of focal ants: $F_{3,48} = 3.33$, $p = 0.028$
Treatment: $F_{1,47} = 10.35$, $p = 0.002$
Colony origin of focal ants x Treatment: $F_{3,48} = 0.28$, $p = 0.841$

Table S3: Correlation between the number or ORco reads and the total number of OR reads. Analysis of Variance on a negative binomial glm, n = 29. Related to Fig. S1.
Full model: OR read sum ~ Colony ID + Orco reads x Treatment

	Df	Deviance	Resid. Df	Resid. Dev	p
NULL			28	124.290	
colony ID	1	0.075	27	124.215	0.784687
treatment	2	0.128	25	124.087	0.937883
number of orco reads	1	83.942	24	40.145	< 2.2e-16
treatment:orco reads	2	11.081	22	29.064	0.003924

Table S4: List of experimental subcolonies

ID	Number of sub-colonies (8-10 ants each)	Sample size for the behavioural experiment (ants removed*)	Number of samples for transcriptomic data	Focal colony	Exposure odour	Test odour
1	3	n_{Ae} = 15 (7)	5	Ae21	Ae34	Ae34
2	3	n_{Ae} = 20 (3)	5	Ae32	Ae34	Ae34
3	1	n_{Ae} = 5 (4)		Ae16	Ae37	Ae37
4	2	n_{ae} = 3 (12)		Ae7	Ae40	Ae40
5	2	n_{Ae} = 13 (3)		Ae34	Ae43	Ae43
6	2	n_{Ae} = 15 (1)		Ae40	Ae49	Ae49
7	2	n_{Ae} = 11 (5)		Ae43	Ae49	Ae49
8	2	n_{Ao} = 6 (0)	5	Ae21	Ao62	Ao62
9	1	n_{Ao} = 4 (0)	4	Ae32	Ao62	Ao62
10	2	n_{Ao} = 3 (11)		Ae21	Ao2	Ao2
11	1	n_{Ao} = 3 (7)		Ae16	Ao2	Ao2
12	2	n_{Ao} = 6 (9)		Ae7	Ao3	Ao3
13	2	n_{Ae} = 13 (3)		Ae34	Pentan	Ae43
14	2	n_{Ae} = 14 (2)		Ae43	Pentan	Ae49
15	2	n_{Ae} = 14 (2)		Ae40	Pentan	Ae49
16	3	n_{Ae} = 17 (2)<br>n_{Ao} = 4 (0)	5	Ae32	Pentan	Ao62<br>Ae34
17	5	n_{Ae} = 16 (2)<br>n_{Ao} = 16 (8)	4	Ae21	Pentan	Ao2<br>Ao62<br>Ae34
18	3	n_{Ae} = 10 (4)<br>n_{Ao} = 7 (7)		Ae7	Pentan	Ao3<br>Ae40
19	2	n_{Ae} = 5 (4)<br>n_{Ao} = 3 (3)		Ae16	Pentan	Ao2<br>Ae37

* numbers in brackets indicate the number of ants removed from data set because they did not contact the slide with the non-nestmate CHC extract

Table S8. The number of ORs per subfamily in the *Acromyrmex echinatior* genome and the number of genes that were affected by the odour treatment. We tested for each subfamily with more than 2 genes separately whether genes of the subfamily were more or less likely to be affected by the habituation treatment using Fisher's exact tests. We tested this once using a list of ORs that were differentially expressed after correcting for false discovery rate (p_{adj} < 0.05). Because of the exploratory nature of this analysis and because we have reason to believe that many genes were affected with only mild effect sizes, we repeated this analysis with all the genes that had an initial p < 0.05 in the Deseq2 analyis. The genes affected by habituation are the union of all genes affected by exposure to either con- or allospecific non-nestmate odours. Note that the effect for subfamily 9E is still significant after Bonferroni-Holm adjustment correcting for testing each subfamily separately (p_{BH} = 0.034), that of family L is not (p_{BH} = 0.31). Related to Fig. 3B.

Subfamily	Number of genes in the genome	Number of genes affected by odour exposure p_{adj} < 0.05	Fisher's exact p value for p_{adj} list	Number of genes affected by odour exposure pvalue < 0.05	Fisher's exact p value for pvalue list
9E	134	8	0.65	66	0.0026
A	10	1	0.44	2	0.74
B	1	1		1	
C	1	0		0	
D	4	0	> 0.99	1	> 0.99
E	35	2	0.71	9	0.59
F	4	0	> 0.99	1	> 0.99
G	1	0		0	
H	16	2	0.23	8	0.35
J	2	0	> 0.99	0	
K	2	0	> 0.99	0	
L	68	1	0.23	12	0.026
M	5	0	> 0.99	0	0.34
N	7	0	> 0.99	0	0.20
O	1	0		0	
Orco	1	0		0	
P	10	0	> 0.99	4	0.76
Q	1	0		0	
R	3	0	> 0.99	0	0.58
S	2	0	> 0.99	0	
T	7	0	> 0.99	2	> 0.99
U	33	1	> 0.99	15	0.39
V	53	3	0.75	12	0.23
W	1	0		0	
XA	1	0		0	
Z	1	0		0	
unknown	32	4	0.10	15	0.30
